# Interfacial Characteristics of 6061/AZ31B Composites in Multi-Pass Rolling

**DOI:** 10.3390/ma15031199

**Published:** 2022-02-04

**Authors:** Haokun Yang, Yuandong Li, Jin Qiu, Zhaoxi Song, Guangli Bi, Hongwei Zhou

**Affiliations:** 1 State Key Laboratory of Advanced Processing and Recycling of Nonferrous Metals, Lanzhou University of Technology, Lanzhou 730050, China; yanghk_0123@163.com (H.Y.); qiujin_ww@163.com (J.Q.); lionel_xi@163.com (Z.S.); glbi@163.com (G.B.); zhouhongwei126ct@126.com (H.Z.); 2 Key Laboratory of Non-Ferrous Metal Alloys and Processing, Ministry of Education, Lanzhou University of Technology, Lanzhou 730050, China

**Keywords:** 6061 aluminum alloy, AZ31B magnesium alloy, multi-pass rolling, interface organization, mechanical property

## Abstract

An Al 6061/Mg AZ31B composite plate with good bonding and excellent comprehensive mechanical properties was prepared through solid-liquid cast-rolling bonding (SLCRB). The microstructure evolution and mechanical behavior of Al/Mg composite plates under different rolling passes were studied. The results showed that with the increase of rolling passes, the bonding layer of the composite plate was crushed, and the base material on both sides of the substrate gradually grew towards the broken part of the bonding layer. The microstructure on both sides of the substrate extended along the rolling direction and was dynamically recrystallized to a certain extent. In the Mg substrate, because the preheating temperature was higher than its recrystallization temperature, with the increase of rolling passes, the grains in Mg substrate were crystallized. When the rolling passes reached the fourth pass, complete recrystallization basically took place in the Mg substrate. With the change of the internal structure and bonding layer on both sides of the substrate, the mechanical properties of the composite plate can be improved gradually. The tensile strength increased from 136 MPa before rolling to 190 MPa at the fourth pass, and the shear strength increased from 74 MPa to 98 MPa, with growth rates of about 40% and 32%, respectively. The elongation of the composite plate decreased from 6.3% to 5.4%, a decrease of about 1%.

## 1. Introduction

In recent years, the energy crisis and environmental pollution in the world have become increasingly serious. Reducing the use of precious metal resources to reduce energy consumption and making the weight of vehicles such as automobiles and airplanes less than before to reduce environmental pollution have become urgent problems for the manufacturing industry. Therefore, the technical development and practical application of lightweight composite materials have become the focus of materials research [1]. Metal matrix composites integrate the advantages of each component in the design, make up for the shortcomings of each component, and have excellent properties unmatched by a single metal or alloy [2]. Wu et al. [3] reported that real solid-state bonding in composites has been achieved through various metals, including mechanical bonding and diffusion bonding. As we all know, magnesium alloy, as the first light metal used in industry, has the advantages of high specific strength, high specific stiffness, good shock absorption, excellent electrical and thermal conductivity, and so on. However, due to the low equilibrium potential, its surface stability is very poor. Therefore, even if the magnesium oxide layer is formed on the surface of magnesium alloy, it still lacks the ability of self-healing and self-passivation after corrosion, and its corrosion resistance is poor [4]. This is one of the reasons that the application of magnesium alloy in the industry is severely limited. Aluminum alloy is another light metal widely used in industry. Due to its high metal activity, aluminum atoms on the surface of the aluminum alloy react easily with the oxygen in the air, producing a dense layer of Al_2_O_3_ films, which can effectively prevent further reaction between the internal aluminum atoms and oxygen atoms; so aluminum has good corrosion resistance at room temperature. At the same time, aluminum alloy has good plasticity at room temperature due to its face-centered cubic crystal structure [5]. Therefore, the composite of aluminum and magnesium can organically combine the advantages of aluminum and magnesium materials, give full play to their characteristics, make up for the shortcomings of a single material in performance [6], and prepare an ideal lightweight composite material with broad application prospects.

So far, there are many processes for manufacturing Al/Mg composites, such as diffusion welding [7,8], friction stir welding [9,10], explosive welding [11,12], gravity composite casting [13,14], lost mode composite casting [15], rolling [16] and solid-liquid casting and rolling combination [17]. Solid-liquid cast-rolling bonding (SLCRB) is a novelty method in which the liquid metal is poured on the surface of the solid metal, and then both are sent to the rolling mill together. This technology combines the composite casting method with the rolling method, which not only solves the problem of too thick of a bonding layer in the process of compound casting, but also solves the problem of too large residual stress in the process of rolling. This method has attracted the attention of many scholars in recent years. Huang et al. [18] pointed out that compared with other forming methods, SLCRB technology is an emerging composite technology with good development prospects, which has the advantages of low cost, low energy consumption, short process, and high production efficiency. However, the intermetallic compound layer generated at the bonding interface is prone to fracture and debonding under stress, which leads to crack propagation along the interface with the extension of stress time. Finally, the connection between the Al layer and Mg layer disappears and the composite plate breaks and fails [19]. This problem can be solved by introducing an appropriate interlayer between the two components of the composite. However, the interfacial bonding properties of Al/Mg composites prepared by introducing an interlayer still can not meet expectations.

Post-deformation treatment is an effective method to improve the interfacial bonding properties of Al/Mg composites. Paramsothy et al. [20] reported that Al/Mg intermetallic compound was not found on the bonding interface of Al/Mg composites prepared by casting and hot co-extrusion in subsequent testing. Liu et al. [21] realized the connection between Al and Mg through composite casting and multi-pass warm diameter rolling. It was found that the brittle intermetallic compound layer would break after multi-pass rolling, which improved the interfacial bonding properties of the composites.

For the production of the composite plate with different thicknesses, the rolling process is not only effective [22], but also can improve the mechanical properties of the composite plate [16]. Zhang et al. [23] found that with the increase of the reduction rate, the grain size and diffusion layer width decrease, and the bonding strength increases with the increase of the reduction rate. At present, there are few reports on using multi-pass rolling subsequent treatment processes to improve the interfacial bonding properties of Al/Mg composites prepared by SLCRB, and the microstructure evolution in the composites during rolling plays a very important role in the preparation of composites. Many as-cast composites (i.e., plates and bars) need further deformation treatment before formal service. In this study, Al 6061/Mg AZ31B composite plate with Zn interlayer was prepared through SLCRB and multi-pass rolling. The purpose was to study the evolution of the matrix and intermetallic compound layer during multi-pass rolling of Al/Mg composite plate after SLCRB and the effect of these changes on the bonding properties of the composite plate. It provides a new way to improve the interfacial bonding properties of composites.

## 2. Materials and Methods

### 2.1. Materials and Preparation Processing

The materials used in this experiment were Al 6061 alloy and Mg AZ31B alloy, and their chemical composition is shown in Table 1. The industrial ingot Al 6061 was used as the cladding metal and the solid substrate Mg AZ31B was used as the rolled plate.

When the voltage was 25 V, the current was 80 A, the spraying distance was 100 mm, the flow rate was 80 L/h, and the spraying thickness was about 100 μm. Under the above parameters, the Zn interlayer was prepared on magnesium alloy solid rectangular plate by arc spraying. After spraying, the magnesium plate was diffused at 633 K (360 °C) for 20 min, and a diffusion layer containing Zn was produced on the Mg substrate, which ensured the good bonding of the material.

**Figure 1:**
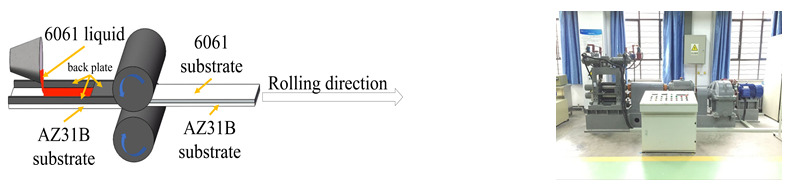
Schematic diagram of SLCRB of 6061/AZ31B composite plate and two-roller hot and cold mill.

As shown in Figure 1, the Al 6061/Mg AZ31B composite plate was produced by the following preparation process: (i) The prepared Al ingot is melted in a 7.5 kW well crucible resistance furnace. When the temperature of molten Al 6061 reaches 993k (720 °C), C_2_Cl_6_ with a melt mass fraction of 1% is used for refining. After holding at 993 K (720 °C) for about 5 min, the melt temperature is adjusted according to the set temperature (953 K (680 °C)). (ii) The metal melt is quantitatively poured on the solid substrate (298 K (25 °C)), and the roll gap width is set to 7 mm. The rolling mill is started, the solid substrate coated with liquid metal is sent to the roll for SLCRB, and then the Al/Mg composite plate is obtained through air cooling. The rolling speed is set to 42 mm/s and the maximum rolling force is set to 420 kN.

The rolling parameters used in this study are shown in Table 2. The roll diameter was 180 mm, the maximum rolling force was 420 kN, and the rolling speed range was 0.2–0.6 m/s. In this study, a rolling speed of 6 rpm was selected to carry out 5 passes of rolling, and the reduction rate of each pass was set as 15% of the previous pass. Before rolling each time, composite materials were placed in a SRJX-4-12 box-type resistance furnace (Cangzhou Ouhai Test Instrument Co., Ltd., Cangzhou, Hebei, China) for thermal insulation at 350 °C for 10 min. On the premise of ensuring that the bonding layer does not diffuse greatly, most of the residual internal stress generated during the previous pass of rolling was eliminated to the greatest extent. After each pass of rolling, the composite plate was air-cooled to room temperature. The coordinated deformation of Al and Mg components was realized and the warpage of the composite was reduced. The actual thickness of the composite was larger than the theoretical thickness due to the spring-back in the rolling process.

### 2.2. Characterization Description

The microstructure of Al, Mg, and intermediate bonding layer in the composite plate was observed by an Axio Scope A1 optical microscope (OM, NIKON instruments, (Shanghai), Co., Ltd., Shanghai, China). The transverse normal (TD-ND) plane was taken as the observation plane. The microstructure, shear section morphology, and chemical composition of the bonding interface were observed by a QUANTUM FEG-450 thermal field emission scanning electron microscope (SEM-EDS, NEC Electronics Corporation, Tokyo, Japan). A high-resolution field emission scanning electron microscope (HRFESEM, TESCAN MAIA3, TESCAN, Brno, Czech) and electron backscattering diffraction (EBSD, TESCAN, Brno, Czech) system were used to study the polar and inverse polar patterns and grain size of composite plates. The EBSD samples were mechanically polished and then polished with a 2.5 kV ion-beam on a Leica RES101 (Leica, Wetzlar, Germany) for 1 h. Finally, the corresponding data were obtained under the condition of 20 kV and step size of 0.2 μm.

### 2.3. Mechanical Property Analysis and Thickness Measurement

**Figure 2:**
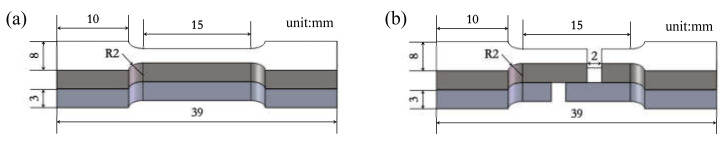
Sample dimensions: (**b**) tensile sample, (**a**) shear sample.

The mechanical properties of the composites were tested by an electronic universal material testing machine (WDW-100D, Jinan Hengxu Testing Machine Technology Co., Ltd., Jinan, China). The tensile test and shear tests were carried out at room temperature at a strain rate of 0.025/min. The sampling direction of the sample was parallel to the rolling direction (RD). Figure 2 shows the specimen sizes required for tensile and shear tests. The shear strength was calculated as follows:

(1) $$\mathrm{averageshearstrength}=\frac{\mathrm{peakload}}{\mathrm{bondwidth}\times \mathrm{bondlength}}$$

The average thickness of each layer was measured by using a vernier caliper after each roll. The measurement was repeated 12 times for each layer. After removing the maximum and minimum values, the average value of the remaining values was taken as the final thickness of each layer.

## 3. Results and Discussion

### 3.1. Effect of Rolling Passes on Microstructure

**Figure 3:**
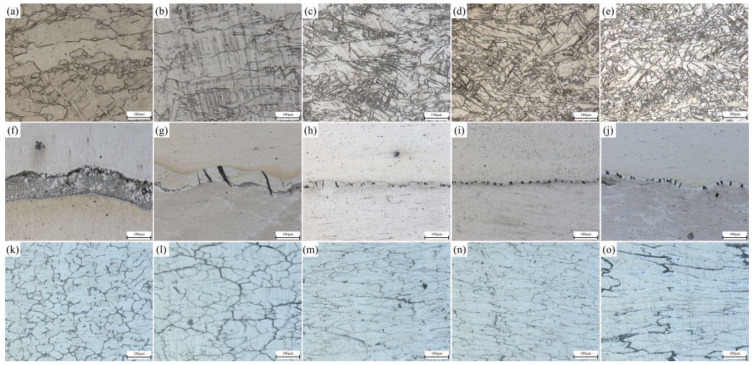
Interface of Al/Mg composite plate under different rolling passes: Mg side: (**a**) not rolled, (**b**) first pass, (**c**) second pass, (**d**) third pass, (**e**) fourth pass; Intermediate bonding layer: (**f**) not rolled, (**g**) first pass, (**h**) second pass, (**i**) third pass, (**j**) fourth pass; Al side: (**k**) not rolled, (**l**) first pass, (**m**) second pass, (**n**) third pass, (**o**) fourth pass.

During the rolling process, the composite pla underwent plastic deformation, which causes the microstructure of the substrate on both sides to extend along the rolling direction. When the deformation temperature reached the dynamic recrystallization temperature of 0.6 Tm (Tm is the melting point) and the plastic deformation exceeded the critical strain (or peak strain) of the substrate material on both sides, plate dynamic recrystallization occurred inside the substrate and new grains were generated, which transformed the tensile grains into equiaxed grains [24]. With the increase of deformation temperature and strain, the volume fraction of plate dynamic recrystallization also increased. When the plastic deformation became large enough, complete plate dynamic recrystallization occurred inside the metal, and the grains become equiaxed [25]. OM photos of the Mg side, bonding layer, and Al side of the composite plate prepared in this experiment are shown in Figure 3. It can be seen that with the increase of rolling passes, the bonding layer was gradually crushed. At the third pass and the fourth pass (Figure 3i,j), both Al and Mg substrates had a tendency to grow towards the bonding layer. From the microstructure of the Mg substrate (Figure 3a–e), it can be seen that with the increase of rolling passes, the grain size in the Mg substrate gradually decreased and was distributed along the rolling direction. When the fourth pass of rolling was carried out, complete plate dynamic recrystallization occurred in the metal. The reasons are as follows: First, Mg alloy has a reduced slip system and dislocation is easy to plug up, which quickly reaches the dislocation density required for dynamic recrystallization; Second, the stacking fault energy of Mg and Mg alloys is low and the extended dislocations are difficult to gather, so it is difficult to slip and climb, and the plate recovery speed is slow, which is conducive to the occurrence of dynamic recrystallization. Third, the grain boundary diffusion rate of Mg alloy is high, and the dislocation accumulated on the subgrain boundaries can be absorbed by these grain boundaries, thus accelerating the process of plate dynamic recrystallization [26]. All these factors resulted in the complete plate dynamic recrystallization of Mg substrate during the fourth rolling. It can be seen from the microstructure of Al substrate (Figure 3k–o) that with the increase of rolling passes, the grains in the Al substrate were gradually elongated and distributed along the rolling direction, and only a few grains were recrystallized. This is because the higher strain rate leads to a large number of dislocation entanglements, which leads to stress concentration and reduces the nucleation rate of plate dynamic recrystallization. A lower temperature would weaken the diffusion rate of vacancy atoms, making it difficult for the cross slip of dislocation to occur, limiting their plate dynamic recrystallization nucleation, and thus improving the critical conditions for plate dynamic recrystallization [27]. It is worth noting that the initial resulting microstructural arrays of Al samples are constituted by grains and dendritic arms. The former had its sizes substantially decreased with the evolution of rolling stages. However, the dendritic spacings were deformed, and after four rolling passes, the average of these arm spacings was maintained. A similar observation was reported when Al-based alloy powders were investigated [28]. These microstructural parameters associated with other ones are responsible for the resulting mechanical strengths [28].

**Figure 4:**
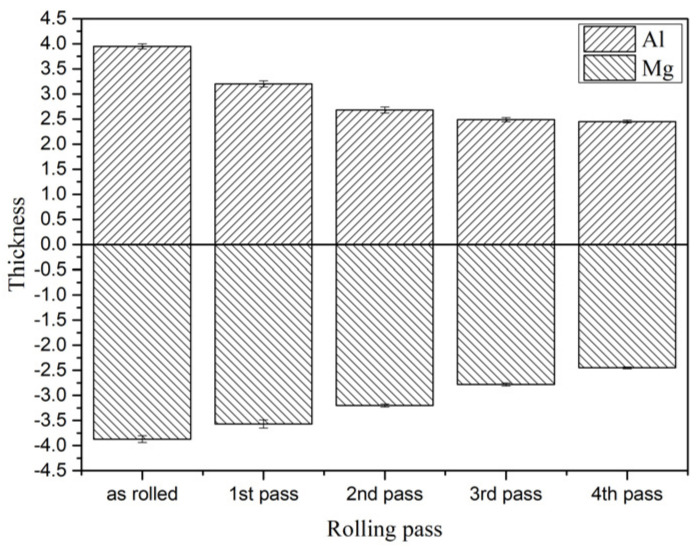
Thickness values of different rolling passes of Al and Mg substrates.

In order to study the coordinated deformation of the 6061/AZ31B composite plate after different passes of rolling, the relative deformation is shown in Figure 4. The contribution of Mg and Al substrates to the reduction rate of the composite plate is summarized in Table 3. It can be seen that the Al substrate bore most of the deformation in the first and second passes of rolling due to the as-cast structure before rolling. The thickness of the Al substrate was significantly reduced. However, during the last two rolling periods, a large amount of energy and deformation accumulated in the Al substrate, and the deformation capacity was obviously weakened. At this time, the Mg substrate bore most of the deformation, and the two substrates achieved coordinated deformation.

### 3.2. Distribution of Alloying Elements in Different Rolling Passes

**Figure 5:**
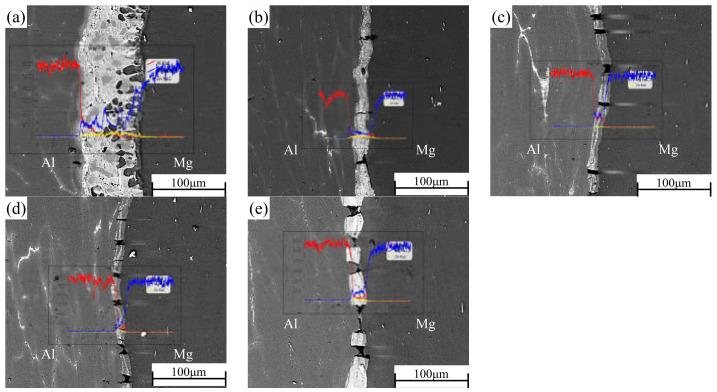
Interface and EDS line scanning results of Al/Mg composite plate under different rolling passes: (**a**) not rolled, (**b**) first pass, (**c**) second pass, (**d**) third pass, and (**e**) fourth pass.

**Figure 6:**
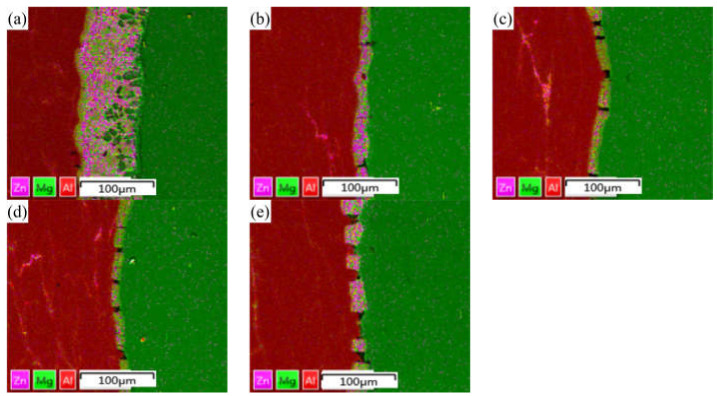
EDS surface scanning results of Al/Mg composite plate interface under different rolling passes: (**a**) not rolled, (**b**) first pass, (**c**) second pass, (**d**) third pass, and (**e**) fourth pass.

In the process of rolling, due to the action of rolling mill pressure, the original bonding layer of the composite plate was gradually crushed, and the substrate on both sides was gradually grown into the cracks of the bonding layer. After rolling, the composite plate was air-cooled to room temperature, which takes about 1 h. EDS was used to scan the bonding part in the middle of the composite plate to determine the element distribution of the bonding interface after each roll. The results of linear scanning are shown in Figure 5. The thickness of the bonding layer decreased gradually from 60 μm to 20 μm at the third pass of rolling. The thickness of the bonding layer after the first and second passes was 35 μm and 24 μm, respectively. The thickness of the bonding layer increased to 25 μm after the fourth pass of rolling. The thickness of the bonded layer decreased the most at the first rolling, and the reduction of the thickness of the bonded layer was slightly larger than the reduction of each pass because the hardness and plasticity of the AlMg_4_Zn_11_ phase in the bonded layer were higher than those of the base metal on both sides (the calculated values have been reported in the previous paper). In the binding region, it can be observed that the concentration of Al decreases from the side of 6061 aluminum alloy to the side of AZ31B magnesium alloy, while the concentration distribution of Mg is the opposite. In the rolling process, severe plastic deformation caused the temperature to rise, and the temperature rise increased with the increase of rolling passes [23]. In this experiment, air cooling was carried out after each roll, and the reduction rate of each roll was set as 15% of the previous one. Therefore, the increase of temperature during rolling does not greatly increase the diffusion coefficient of Al and Mg, resulting in the growth of the interfacial bonding layer. However, with the increase of rolling passes (i.e., the increase of reduction rate), the diffusion coefficient and cooling time will increase with the increase of reduction rate. Accordingly, after the fourth pass of rolling, the width of the diffusion layer increased with the increase of rolling temperature caused by the rolling reduction rate. From the surface scanning results in Figure 6, it can be seen that the Zn element is mainly distributed in the intermetallic compound of the interface bonding layer, and large-scale diffusion occurs to the substrates on both sides.

### 3.3. EBSD Analysis of Different Rolling Passes

**Figure 7:**
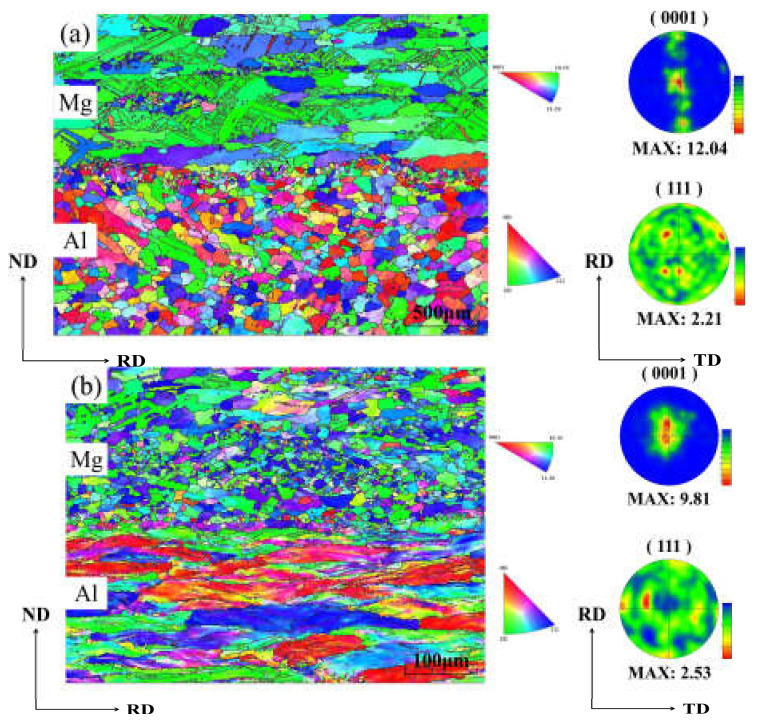
EBSD test results of Al and Mg composite plates: (**a**) not rolled, (**b**) fourth pass.

The effect of rolling pass on the texture evolution of the Al/Mg composite plate was studied by the EBSD method. Figure 7 shows the reverse pole diagram (IPF) and pole diagram (PF) of the Al/Mg composite plate under unrolled conditions and four-pass rolling conditions. After four passes of rolling, most Al grains were in a long strip shape along RD, indicating that it was difficult to stimulate the recovery and dynamic recrystallization of Al substrate at 350 °C for 10 min. Therefore, the (111) PFs of Al substrate exhibited a typical {112} < 111 > copper texture, which is relatively stable and prevalent in face-centered cubic (FCC) metal plates, especially in FCC metals with high stacking fault energy like Al [29]. The copper texture strength increased from 2.11 to 2.53 with the increase of rolling passes to four passes. On the whole, only part of the grains near the bonding zone recrystallized in the Mg substrate before rolling, and the texture disappeared, while most of the texture remained in the Mg substrate. When the composite plate was rolled four times, the Mg substrate was dominated by equiaxed grains, indicating that sufficient dynamic recrystallization took place in the Mg substrate, and the original texture and microstructure of the Mg substrate recrystallized to form fine equiaxed grains. AZ31 grains were held at 350 °C for 10 min and completely recrystallized under four passes of rolling because of the influence of strong internal and external stress [30]. In addition, from the PFs of the Mg substrate after four passes of rolling, it can be seen that all (0001) substrate textures are stretched along RD. The texture strength decreased from 12.04 before rolling to 9.81 after four passes of rolling because a large number of dynamic recrystallizations occurred in the Mg substrate, and the original texture in Mg substrate disappeared, resulting in the decline of texture strength.

**Figure 8:**
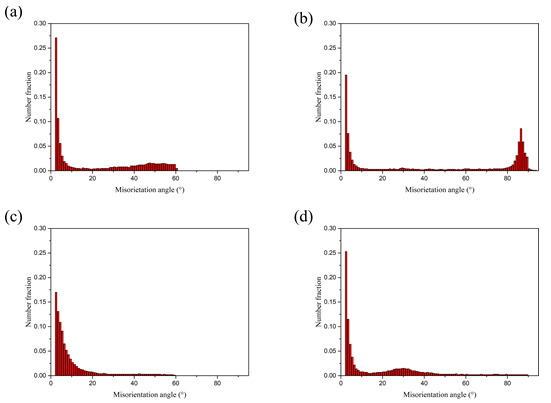
Grain boundary dislocation of Al/Mg composite plate: not rolled: (**a**) Al, (**b**) Mg; The fourth pass: (**c**) Al, (**d**) Mg.

Figure 8 shows the distribution results of directional Angle of Al and Mg substrates after no rolling and four passes rolling, and the corresponding directional Angle distribution also show the same results as above. The grain boundary Angle of Mg grains after four passes of rolling is about 30°, which is closely related to the grain boundary Angle of recrystallized Mg grains.

**Figure 9:**
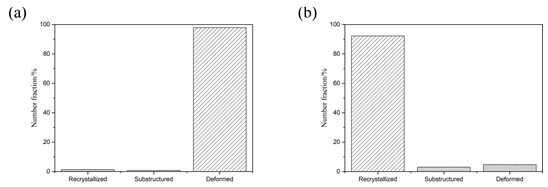
Diagram of microstructure, recrystallization, and large deformation grain number of Al/Mg composite plate: (**a**) Al, (**b**) Mg.

From Figure 9 after four times of rolling Al substrate and Mg substrate central organization, large deformation and dynamic grain size recrystallization can be seen in the Al substrate in the vast majority of grains. In contrast, for the Mg substrate, only a small degree of dynamic recrystallization of the grain size was observed, in accordance with the above analysis. 

### 3.4. Variation of Mechanical Properties with Rolling Passes

**Figure 10:**
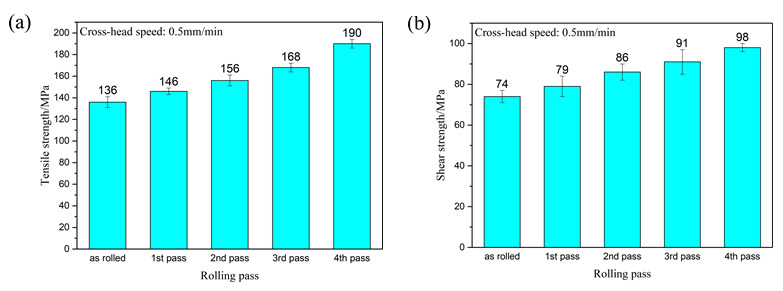
Tensile strength (**a**) and shear strength (**b**) of Al/Mg composite plate under different rolling passes.

In this experiment, three tensile and shear samples were taken from the samples produced after each pass of rolling, and the sample sizes are shown in Figure 2. A tensile test and shear test at room temperature were carried out at a speed of 0.5 mm/min. The average of the values measured by the three experimental data was taken as the final experimental data under the experimental parameters, and the error value between the measured data and the final experimental data was represented by an error bar. The tensile strength and shear strength of the composite plate prepared by four passes are shown in Figure 10.

**Figure 11:**
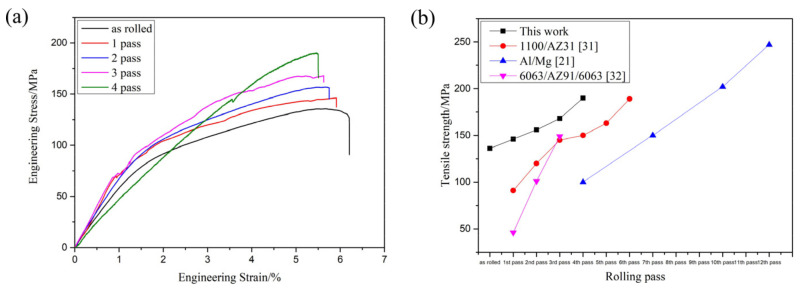
(**a**) Tensile stress-strain curve of Al/Mg composite plate at room temperature in different rolling passes, (**b**) Relevant scholars’ tensile strength data of Al/Mg composite plate [21,31,32].

Figure 10a shows the tensile strength of the composite plate prepared by four passes. It can be seen that the tensile strength of the composite plate increased gradually with the increase of rolling passes. When rolling to the fourth pass, the tensile strength of the composite plate increased by about 40% compared with that of the unrolled plate. This is because the grains in the metal substrates on both sides were deformed and recrystallized during rolling, and the mechanical properties of the deformed grains along the rolling direction were greatly improved. After dynamic recrystallization, the grain size was far smaller in volume than before. This dynamic recrystallization strengthened the fine grain, greatly increasing the tensile strength of the substrate. With the increase of rolling passes, the tensile strength of the composite plate was gradually increased, but at the expense of the elongation of the composite plate. As shown in the stress-strain curve in Figure 11a (Some relevant data are shown in Table 4), with the increase of rolling passes, the tensile strength of the composite plate increased from 136 MPa (unrolled plate) to 190 MPa (fourth pass plate), and the tensile strength increased by about 40%. The elongation of the composite plate decreased from 6.3% to 5.4%, and the elongation decreased by about 1%.

Figure 11b shows the relevant data of tensile strength obtained by relevant scholars from the research on rolling passes of Al/Mg composites. It can be seen that the tensile strength of Al/Mg composites increases with the increase of rolling passes because grain deformation and dynamic recrystallization occur in the base material during the rolling of Al/Mg composites. All of these will improve the tensile properties of composites.

**Figure 12:**
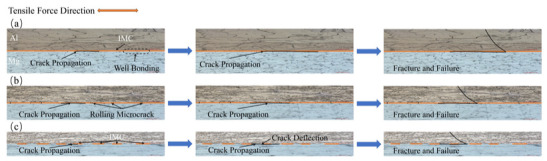
Schematic diagram of crack propagation: (**a**) not rolled, (**b**) second pass, (**c**) fourth pass.

The schematic diagram of crack propagation is shown in Figure 12. In Figure 12a, the composite plate is not rolled and the bonding layer is in a continuous state. During the shear test, the interfacial bond layer was composed of brittle and hard intermetallic compound phases. With the application of tensile force, cracks appeared in the interfacial bond layer. As tension increased, the crack increased gradually. However, because the crack direction and the shear stress direction were parallel, these cracks could propagate directly. Then, in the area where the bonding between the interface and the matrix was weak, the interface bond layer was stratified and the crack propagated along with the interface. After that, the cracks generated in the bonding layer were connected to form a larger one. With the progress of the tensile test, when the tensile force was large enough, the crack expanded rapidly along with the interface and passed through to the other side at an angle of 45° in the Mg substrate and reached the other side of the interface. At this point, the Al and Mg substrates separated. The composite panels failed completely. In Figure 12b, the interlayer has been fractured due to rolling and is no longer in a continuous state. With the application of tension, cracks appeared in the interfacial bonding layer. As the tension increased, the crack expanded along with the interface, and when the crack expanded to the crack on the bonding interface, part of the stress accumulated at the crack tip was released. In the part without separation, new cracks were generated as the tension continues to increase, and the above process was repeated again. When the tension increased to a certain extent, the cracks penetrated the Mg layer and fractured the Mg substrate, resulting in composite plate failure. In Figure 12c, due to the large reduction rate, the base materials in the Al and Mg substrates on both sides are squeezed into the cracks in the bonding layer and placed into contact with each other, forming an alternate structure of hard and soft. During the tensile test, cracks were initially apparent in the intermetallic compound at the Al/Mg interface. With the increase in the tensile crack along with weakened regional extension of the layer binding force, when it extended to the interface of Al and Mg, the crack changed in the direction of the deflection. When the intermetallic layer was extended again, another deflection occurred due to different binding forces. When the tensile strain reached 5.4%, the crack passed through the whole composite plate and the composite plate failed in stratification. In conclusion, in the tensile test of the Al/Mg composite plate in which the middle layer was crushed and both sides of the base metal contacted each other, the well-bonded interface layer blocked the crack propagation and absorbed energy due to uniform fracture and crack deflection, thus improving the bonding strength. This is consistent with what Wang et al. [19] reported.

Figure 10b shows the shear strength of the composite plate prepared by four passes. It can be seen that the shear strength of the composite plate increased gradually with the increase of the rolling passes. When rolling to the fourth pass, the shear strength of the composite plate increased about 32% compared with the tensile strength of the unrolled plate. This is because with the increase of rolling passes, the bonding layer was cracked, and the base metal in the substrates on both sides was gradually squeezed into the crack of the bonding layer. At this time, the bonding layer became a soft and hard phase structure composed of the intermetallic compounds with low plasticity and high stiffness and base metal with high plasticity and low hardness. When the crack propagated, the crack propagation direction changed.

### 3.5. Shear Section Analysis

**Figure 13:**
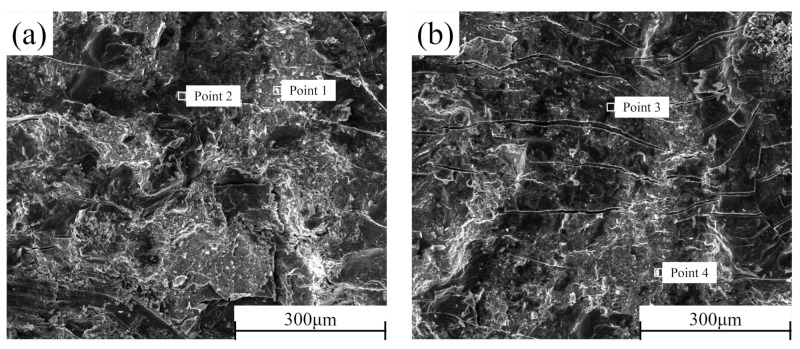

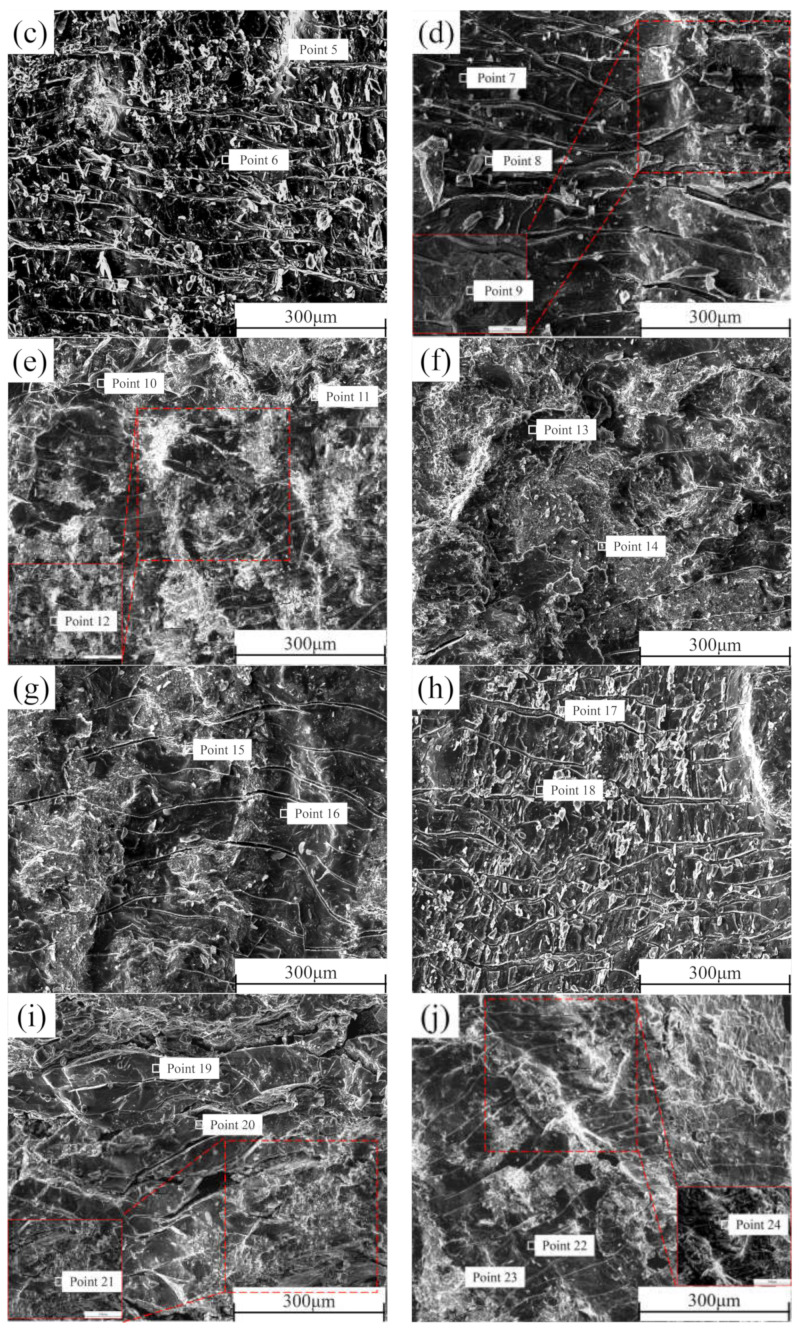
SEM cross-section of the composite plate under different rolling passes: Al side: (**a**) not rolled, (**b**) first pass, (**c**) second pass, (**c**) third pass, (**d**) fourth pass; Mg side: (**f**) not rolled, (**g**) first pass, (**h**) second pass, (**i**) third pass, (**j**) fourth pass.

In order to better determine the failure mechanism of the middle layer of the composite plate, the cross-sections of the shear samples prepared before rolling and after each pass of rolling were detected by SEM-EDS. The section morphology is shown in Figure 13. It can be seen that there is no macroscopic plastic deformation trace on the section of the composite plate when it is not rolled. The fracture surface is smooth and distributed. With the increase of rolling passes, many parallels and continuous “river-like” cracks appeared on both sides of Al and Mg, and many small planes (facets) existed at the same time. From the first pass to the second pass, the number of “river-like” cracks on both sides of Al and Mg sections continued to increase. When the rolling passes increased to the third pass and the fourth pass, the number of “river-like” cracks on the section decreased, and ridge-like cracks appeared. This is because, with the increase of rolling passes, the reduction rate and the increase of rolling temperature caused by the increase of the reduction rate encourage the metal substrate on both sides to grow towards the crack at the bonding layer, resulting in the self-healing process of the composite plate [33]. Combined with the EDS point scanning detection results (Table 5), it can be seen that Zn content is relatively high in the brighter part of the SEM image. According to the atomic calculation ratio detected by EDS, it can be estimated that the Al cross sections before rolling after the first rolling, and the second rolling contain three phases: Al3Mg2, Al12Mg17, and AlMg4Zn11. All point scanning results show that the atomic percentage of the Al element is less than 63%. Therefore, it can be inferred that the Al matrix did not appear on these three sections, and the fracture occurred at the bonding layer of the composite plate. However, in the third pass of rolling and the ridged crack on the section after the fourth pass of rolling, the atomic percentage of Al element was as high as 98%, indicating that in the two passes of rolling, the Al side matrix had grown to the binding layer, which was consistent with the surface scanning results in Section 3.2. Similarly, this phenomenon can also be observed on the Mg side section. In contrast to the Al side, the presence of Al3Mg2 phase was not detected on the Mg side section, indicating that the toughness of the Al3Mg2 phase was higher than that of the Al12Mg17 phase, and the fracture occurred in the Al12Mg17 binding region with poor performance, which is consistent with the previous research [34].

## 4. Conclusions

Through the study on the plate thickness ratio, microstructure, and bonding strength of Al 6061/Mg AZ31B composite plate containing a Zn interlayer after different rolling passes, the following conclusions are obtained:

1. The Al/Mg composite plate bonding layer is crushed by multi-pass rolling, and the base material on both sides of the substrate gradually grows towards the fracture of the bonding layer.
2. With the increase of rolling passes, the grains in the 6061 substrate grow into strips along the rolling direction. The preheating temperature and deformation amount are not enough to cause dynamic recrystallization, and most of the internal grains are large deformation grains.
3. The internal grains of AZ31B substrate change from the initial rolling state to fine equiaxed grains and the melting point of AZ31B is lower than 6061. Under the same conditions, dynamic recrystallization occurs in the AZ31B substrate, and the internal grains are basically recrystallized after the fourth pass of rolling.
4. The tensile strength and shear strength of the Al/Mg composite plate containing the Zn interlayer can be improved by multi-pass rolling. With the increase of rolling passes, the tensile strength and shear strength of the Al/Mg composite plate increase, and the elongation decreases.
5. After the fourth pass of rolling, the tensile strength and shear strength of the composite plate reaches the maximum values, which are 190 MPa and 90 MPa, respectively. The elongation was 5.4%.

## Figures and Tables

**Table 1:** Chemical constituents of 6061 Al alloy and AZ31B Mg alloy.

Material	Mass Fraction/%						
	Al	Mg	Zn	Mn	Si	Cu	Fe
Al 6061	Bal.	0.90	0.02	0.02	0.63	0.26	0.11
Mg AZ31B	2.66	Bal.	0.73	0.44	0.01	-	-

**Table 2:** Theoretical and practical scheme of rolling process parameters.

Rolling Pass	Plate Thickness before Rolling/mm	Theoretical Thickness after Rolling/mm	Actual Thickness after Rolling/mm	Theoretical Reduction/mm (Reduction Rate)	Actual Reduction/mm (Reduction Rate)
1	7.82 ± 0.06	6.65	6.77 ± 0.07	1.17 (15%)	1.05 (13.43%)
2	6.77 ± 0.07	5.75	5.88 ± 0.04	1.02 (15%)	0.89 (13.15%)
3	5.88 ± 0.04	5.00	5.27 ± 0.02	0.88 (15%)	0.61 (10.37%)
4	5.27 ± 0.02	4.48	4.90 ± 0.02	0.79 (15%)	0.37 (7.02%)
5	4.90 ± 0.02	4.17	Broken	0.73 (15%)	Broken

**Table 3:** Contribution of Al and Mg to a reduced rate of composite plate.

Rolling Pass	Thickness of Mg Substrate before Rolling/mm	Thickness of Mg Substrate after Rolling/mm	Reduction Contribution Ratio/%	Thickness of Al Substrate before Rolling/mm	Thickness of Al Substrate after Rolling/mm	Reduction Contribution Ratio/%	Actual Total Reduction/mm
1	3.87 ± 0.07	3.57 ± 0.08	28.57	3.95 ± 0.05	3.2 ± 0.06	71.43	1.05
2	3.57 ± 0.08	3.2 ± 0.03	41.57	3.2 ± 0.06	2.68 ± 0.06	58.43	0.89
3	3.2 ± 0.03	2.78 ± 0.03	68.85	2.68 ± 0.06	2.49 ± 0.04	31.15	0.61
4	2.78 ± 0.03	2.45 ± 0.02	89.19	2.49 ± 0.04	2.45 ± 0.03	10.81	0.37

**Table 4:** The YS, UTS, and elongation of each sample in Figure 9a.

	Not Rolled	First Pass	Second Pass	Third Pass	Forth Pass
YS/MPa	12.878	12.609	14.983	15.639	10.669
UTS/MPa	135.669	146.233	156.507	167.883	190.311
Elongation/%	6.275	5.911	5.712	5.577	5.445

**Table 5:** EDS point scanning analysis of composite plate section in different rolling passes.

Area No.	Element Composition, Atomic Fraction/%			Probable Phase
	Al	Mg	Zn	
1	31.5	51.4	17.1	Al_12_Mg_17_ + AlMg_4_Zn_11_
2	64.1	35.2	0.7	Al_3_Mg_2_
3	62.6	37.0	0.4	Al_3_Mg_2_
4	33.5	52.4	14.1	Al_12_Mg_17_ + AlMg_4_Zn_11_
5	28.8	56.9	14.3	Al_12_Mg_17_ + AlMg_4_Zn_11_
6	61.7	38.0	0.3	Al_3_Mg_2_
7	51.2	44.8	4.0	Al_3_Mg_2_
8	29.4	56.0	14.6	Al_12_Mg_17_ + AlMg_4_Zn_11_
9	98.1	1.6	0.3	α-Al
10	55.2	44.9	0.9	Al_3_Mg_2_
11	38.7	50.7	10.6	Al_12_Mg_17_ + AlMg_4_Zn_1_
12	97.9	2.1	0.0	α-Al
13	40.7	55.6	3.7	Al_12_Mg_17_
14	27.3	55.5	17.3	Al_12_Mg_17_ + AlMg_4_Zn_11_
15	14.3	51.3	34.4	Al_12_Mg_17_ + AlMg_4_Zn_11_
16	44.9	51.2	4.0	Al_12_Mg_17_
17	34.4	51.3	14.3	Al_12_Mg_17_ + AlMg_4_Zn_11_
18	46.0	53.5	0.5	Al_12_Mg_17_
19	43.9	55.7	0.4	Al_12_Mg_17_
20	25.3	54.6	20.1	Al_12_Mg_17_ + AlMg_4_Zn_11_
21	8.2	91.8	0.0	α-Mg
22	46.6	53.1	0.3	Al_12_Mg_17_
23	20.1	54.6	25.3	Al_12_Mg_17_ + AlMg_4_Zn_11_
24	5.2	94.6	0.2	α-Mg

## Data Availability

Not applicable.
